# 9 Differential Coagulation Factor Activity in Obese vs. Non-Obese Burn Injured Patients

**DOI:** 10.1093/jbcr/irae036.009

**Published:** 2024-04-17

**Authors:** Shane Mathew, Desiree Pinto, Bonnie C Carney, Lauren T Moffatt, Jeffrey W Shupp, Shawn Tejiram

**Affiliations:** MedStar Washington Hospital Center, Washington, DC; MedStar Health Research Institute, Washington, District of Columbia; Firefighters’ Burn and Surgical Research Laboratory, Washington, DC; Georgetown University School of Medicine, Washington, DC; MedStar Washington Hospital Center, Washington, DC; MedStar Health Research Institute, Washington, District of Columbia; Firefighters’ Burn and Surgical Research Laboratory, Washington, DC; Georgetown University School of Medicine, Washington, DC; MedStar Washington Hospital Center, Washington, DC; MedStar Health Research Institute, Washington, District of Columbia; Firefighters’ Burn and Surgical Research Laboratory, Washington, DC; Georgetown University School of Medicine, Washington, DC; MedStar Washington Hospital Center, Washington, DC; MedStar Health Research Institute, Washington, District of Columbia; Firefighters’ Burn and Surgical Research Laboratory, Washington, DC; Georgetown University School of Medicine, Washington, DC; MedStar Washington Hospital Center, Washington, DC; MedStar Health Research Institute, Washington, District of Columbia; Firefighters’ Burn and Surgical Research Laboratory, Washington, DC; Georgetown University School of Medicine, Washington, DC; MedStar Washington Hospital Center, Washington, DC; MedStar Health Research Institute, Washington, District of Columbia; Firefighters’ Burn and Surgical Research Laboratory, Washington, DC; Georgetown University School of Medicine, Washington, DC

## Abstract

**Introduction:**

Obesity confers a prothrombotic state through an imbalance in hemostasis due to chronic inflammation and inhibition of the fibrinolytic pathway, and it may worsen the coagulopathy seen in burn patients. There is a paucity of data on the role obesity plays in burn injury and controversy exists with some literature indicating a survival benefit termed the “obesity paradox.” This analysis aims to define whether obesity promotes a hypercoagulable state in burn patients through measurement of coagulation factor activity at admission with extrinsic pathway factors expected to be elevated.

**Methods:**

Burn patients admitted to a regional burn center between 2012-2017 were prospectively enrolled and patient demographics, burn injury characteristics, and blood were collected within two hours of admission. Assays were performed to measure the activity of Factors ll, V, Vll, Vlll, lX, X, Xl, and Xll and concentration of Factor lb. To identify differences, patients were stratified into two groups: non-obese (BMI 18.5-29.9 kg/m2) and obese (BMI >30kg/m2). Comparisons were made with the t-test or Mann-Whitney test and chi-square or Fisher’s exact test as appropriate.

**Results:**

There were 129 patients included in the analysis. Of those included, 64% were male with an average age of 42.0 ± 1.4 years and total body surface area (TBSA) burn of 22.5% ± 1.9%. There were no significant differences in age, sex, and percent TBSA between the non-obese and obese groups. While PTT was not different between the non-obese and obese groups (29.59 ± 0.72 vs 27.4 ± 0.58, p=0.18), INR was significantly higher in non-obese patients (1.18 ± 0.03 vs 1.07 ± 0.01, p=0.005), although both groups remained within normal range. Factor IX activity level was above the normal range in both groups and significantly different between non-obese and obese patients (150.4% ± 4.7% vs 183.0% ± 9.6%, p=0.002). Factors Vlll (330.2% ± 14.4% vs 318.6% ± 26.5%, p=0.47) and XI (125.4% ± 5.0% vs 136.5% ± 9.1%, p=0.12) were above normal factor activity levels and Factor VIII was almost double normal levels in both groups. Factors lb, ll, V, Vll, X, and Xll were within normal factor activity, but lb, ll, Vll, and X were significantly higher in the obese group.

**Conclusions:**

Burn injury promotes an early prothrombotic response and produces differential levels of factor activity following injury. Factor IX was significantly higher in obese patients compared to non-obese patients while Factors VIII and XI were elevated in all patients. Classic coagulation measures such as PT, PTT, and INR remain ineffective in determining specific coagulopathies following burn injury. Further study will be necessary to examine the relationship obesity plays in burn induced coagulopathy.

**Applicability of Research to Practice:**

Examination of the impact of obesity on burn induced coagulopathy may identify targets for future interventions.

